# Feasibility and Usability of an Artificial Intelligence—Powered Gamification Intervention for Enhancing Physical Activity Among College Students: Quasi-Experimental Study

**DOI:** 10.2196/65498

**Published:** 2025-03-24

**Authors:** Yanan Gao, Jinxi Zhang, Zhonghui He, Zhixiong Zhou

**Affiliations:** 1 School of Physical Education and Sport Science, Fujian Normal University, Fuzhou, China; 2 Institute of Artificial Intelligence in Sports, Capital University of Physical Education and Sports, 11 Beisanhuan West Road, Beijing, 100191, China, 86 13552505679; 3 The Department of Physical Education, Peking University, Beijing, China

**Keywords:** physical activity, gamification, artificial intelligence, digital health, digital intervention, feasibility study

## Abstract

**Background:**

Physical activity (PA) is vital for physical and mental health, but many college students fail to meet recommended levels. Artificial intelligence (AI)-powered gamification interventions through mobile app have the potential to improve PA levels among Chinese college students.

**Objective:**

This study aimed to assess the feasibility and usability of an AI-powered gamification intervention.

**Methods:**

A quasi-experimental study spanning 2 months was conducted on a sample of college students aged 18 to 25 years old from 18 universities in Beijing. PA data were recorded using the ShouTi Fitness app, and participant engagement was evaluated through surveys. User satisfaction was gauged through the System Usability Scale, while the intervention’s feasibility was assessed through Spearman rank correlation analysis, Mann-Whitney tests, and additional descriptive analyses.

**Results:**

As of July 2023, we enrolled 456 college students. In total, 18,073 PA sessions were recorded, with men completing 8068 sessions and women completing 10,055 sessions. The average PA intensity was 7 metabolic equivalent of energy (MET)s per session. Most participants preferred afternoon sessions and favored short-duration sessions, with men averaging 66 seconds per session and women 42 seconds. The System Usability Scale score for the intervention based on app is 65.2. Users responded positively to the integration of AI and gamification elements, including personalized recommendations, action recognition, smart grouping, dynamic management, collaborative, and competition. Specifically, 341 users (75%) found the AI features very interesting, 365 (80%) were motivated by the gamification elements, 364 (80%) reported that the intervention supported their fitness goals, and 365 (80%) considered the intervention reliable. A significant positive correlation was observed between the duration of individual PA and intervention duration for men (ρ=0.510, *P*<.001), although the correlation was weaker for women (ρ=0.258, *P*=.046). However, the frequency of PA declined after 35 days.

**Conclusions:**

This study provides pioneering evidence of the feasibility and usability of the AI-powered gamification intervention. While adherence was successfully demonstrated, further studies or interventions are needed to directly assess the impact on PA levels and focus on optimizing long-term adherence strategies and evaluating health outcomes.

## Introduction

### Background

Physical activity (PA) is crucial for maintaining both physical and mental health. It plays a significant role in preventing chronic diseases, improving cardiovascular health, and building strength and endurance [1]. For college students, regular PA is especially important as it can alleviate academic stress, enhance cognitive function, and contribute to overall well-being [2]. Despite these benefits, many college students adopt sedentary lifestyles, often due to increased academic pressures, social activities, and time constraints, and time constraints following their transition to college life [3]. This global trend of sedentary behavior poses a significant public health challenge. In China, national surveys reveal that many students fail to meet the recommended 150 minutes of moderate PA per week [4]. A 2021 national survey further highlighted that 12.94% of students from “Double First-Class” universities failed physical fitness standards, while 14.73% were classified as overweight and 5.5% as obese [5]. Addressing these issues is crucial for promoting students’ health and well-being.

Various interventions have been developed to address these concerns, including traditional methods [6-8], digital approaches [9-11], and gamification strategies [11-13]. Traditional interventions typically include structured PA programs, workshops, and physical education classes. While these methods have shown positive outcomes in increasing PA levels and improving metrics like cardiovascular fitness and body composition [14], they often struggle with maintaining long-term participant engagement [15]. Digital interventions, which include mobile apps, web-based platforms, and wearable devices, have become increasingly popular. These tools offer features such as activity tracking, reminders, goal setting, and personalized feedback, which enhance motivation and adherence [16]. However, many existing PA apps and wearables are not optimized for efficiency or comprehensive assessment, and they often fail to fully engage users over time [17].

Gamification interventions, which incorporate game design elements like points, levels, badges, and leaderboards into nongame contexts, have emerged as an effective strategy to increase PA participation [18]. These interventions aim to make PA more enjoyable and engaging, thereby enhancing motivation and participation. Popular among young people, gamification has been shown to increase PA frequency, intensity, and participation, particularly when multiple game elements are used simultaneously [19,20]. However, more research is needed to identify the optimal combination of gamification elements for promoting PA. The field of gamification in PA interventions is still developing, and further advancements are required [21].

The integration of artificial intelligence (AI) into digital interventions holds significant promise. AI-powered solutions can personalize user experiences, enhance engagement, and improve adherence by analyzing user data (eg, activity levels, preferences, and behavior patterns) to tailor content, set realistic goals, and provide feedback [21,22]. Despite these benefits, research on combining AI and gamification in PA interventions is still in its infancy. Most studies in this field focus on machine learning techniques and are limited in scope and quality [23]. A recent scoping review identified AI-powered digital solutions, such as recommendation systems and conversational agents, as key components in PA interventions, but few studies have explored the combination of AI with gamification elements [24]. In addition, most existing research focuses on adults with chronic conditions [25] or children [26], leaving a gap in research targeting college students, a population that could benefit greatly from innovative PA interventions.

### Objective

This study aimed to evaluate the feasibility and usability of an AI-powered gamification intervention based on the ShouTi Fitness app to improve PA levels among Chinese college students. By assessing user experience, engagement, and the intervention’s impact on PA frequency and intensity, this study seeks to provide valuable insights into the potential of integrating AI and gamification to promote healthier lifestyles in this population. Through this investigation, we aim to fill existing gaps in the literature by offering a personalized and engaging solution for increasing PA among college students.

## Methods

### Study Design

This study employed a quasi-experimental design without a control group to assess the feasibility and usability of an AI-powered gamification intervention that includes personalized recommendations, team collaboration and competition, reward systems, and challenge mechanisms.

### Participants

**Figure 1.**
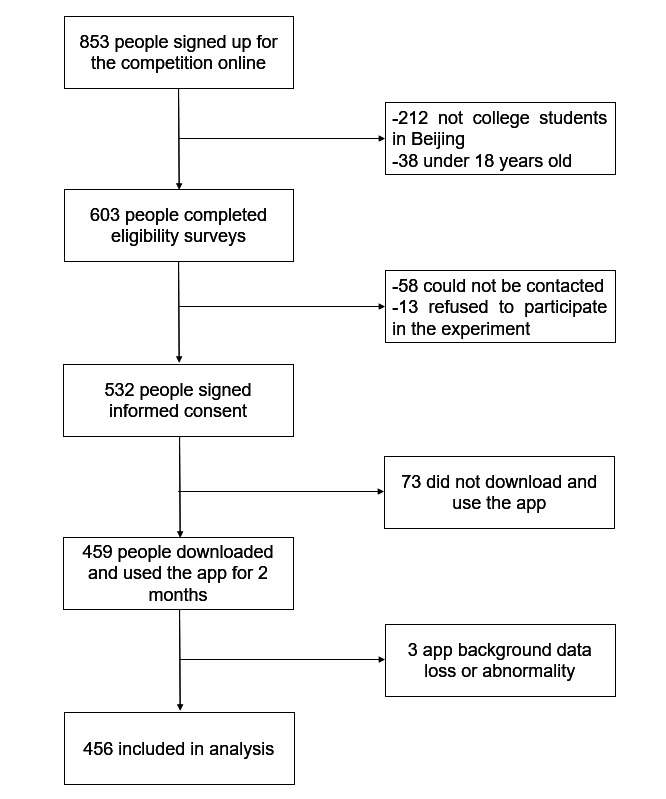
Participant flowchart.

Participants for the study were recruited through advertisements for the “Beijing Student AI Physical Fitness Competition”, which were posted on the official websites of the 18 participating universities and on the ShouTi Fitness app. The recruitment process lasted for 1 month. Prospective participants expressed initial interest by responding to these advertisements. Eligibility was then determined based on their age, enrollment in universities in Beijing and their residence within university premises. Eligible individuals received informed consent forms that outlined the study’s purpose, potential risks, and benefits. Consent was obtained both in person and through email, and participants provided their contact information for follow-up purposes. Participants were excluded from the study if they had missing or abnormal PA data recorded in the app, ensuring the reliability and accuracy of the collected data. The final sample comprised 456 college students (Figure 1), aged 18 to 25 years old, with 239 men and 217 women.

Eligible individuals received informed consent forms that outlined the study’s purpose, potential risks, and benefits. Consent was obtained both in person and via email, and participants provided their contact information for follow-up purposes.

Participants were excluded from the study if they had missing or abnormal physical activity (PA) data recorded in the app, ensuring the reliability and accuracy of the collected data.

The final sample comprised 456 college students (Figure 1), aged 18 to 25 years old, with 239 men and 217 women.

### Procedures

Participants who agreed to take part in the study provided written consent. Upon completing these initial steps, participants were instructed to download and register the ShouTi Fitness app. After successful registration, participants selected their university and joined the “Beijing Student AI Physical Fitness Competition,” where they were randomly assigned to teams based on their university affiliation.

The data collection phase of the study began on May 1, 2023, and concluded on June 30, 2023, spanning a total of 2 months. During this period, participants engaged in an AI-powered gamification intervention using the ShouTi Fitness app. Participants had the autonomy to manually start and stop the app. Throughout the study, participants seamlessly balanced their daily academic responsibilities and personal commitments while using the app. This flexibility allowed them to engage in PA at times that best fit their schedules, supporting a more adaptable approach to increasing PA.

### Intervention

#### ShouTi Fitness App Overview

**Figure 2.**
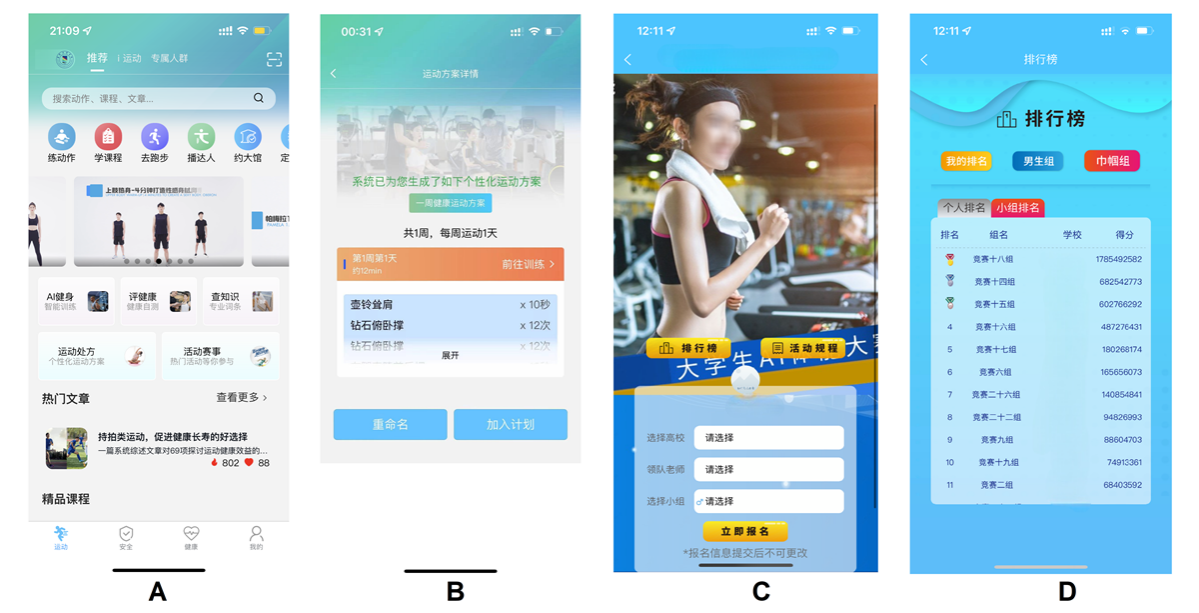
ShouTi fitness interface screenshot.

ShouTi Fitnessis a free app available for both Android and iOS devices, (Figure 2A), designed to serve as part of the AI-powered gamification intervention. The app leverages AI to assess exercise capabilities and generate personalized exercise prescription that align with users’ fitness levels and goals (Figure 2B). The app provides the latest fitness trends, tips, and equipment reviews, and includes tools to track exercise frequency, intensity and duration. In addition, ShouTi Fitness has a built-in exercise risk screening feature to help users identify potential risks and a community interaction function that allows users to connect, share experiences, and motivate each other. All data are securely managed to protect user privacy. The ShouTi Fitness app was developed using principles from Self-Determination Theory, Goal-Setting Theory, and Behavior Change Techniques, with a focus on intrinsic motivation, personalized goals, and sustained engagement. Gamification elements such as team collaboration, dynamic goals, and self-monitoring were designed to meet users’ psychological needs and promote long-term PA.

#### AI-Powered Gamification Intervention

The ShouTi Fitness app integrates a variety of AI- and gamification-powered features designed to optimize PA engagement, enhance motivation, and personalize the user experience. Below are the key components of the AI-powered gamification intervention:

##### Personalized Exercise Recommendations

The main purpose of personalized exercise recommendations is to ensure that users are engaging in targeted and goal-oriented exercise that is effective in meeting their fitness objectives. By receiving daily personalized exercise recommendations, participants are more likely to stay motivated and maintain consistency (Figure 2B). This personalized approach promotes the achievement of individual fitness goals while simultaneously contributing to the overall progress of their teams. As each user completes their personalized workout plan, the team’s collective performance improves, which directly influences their rankings and engagement in the gamification intervention.

**Figure 3.**
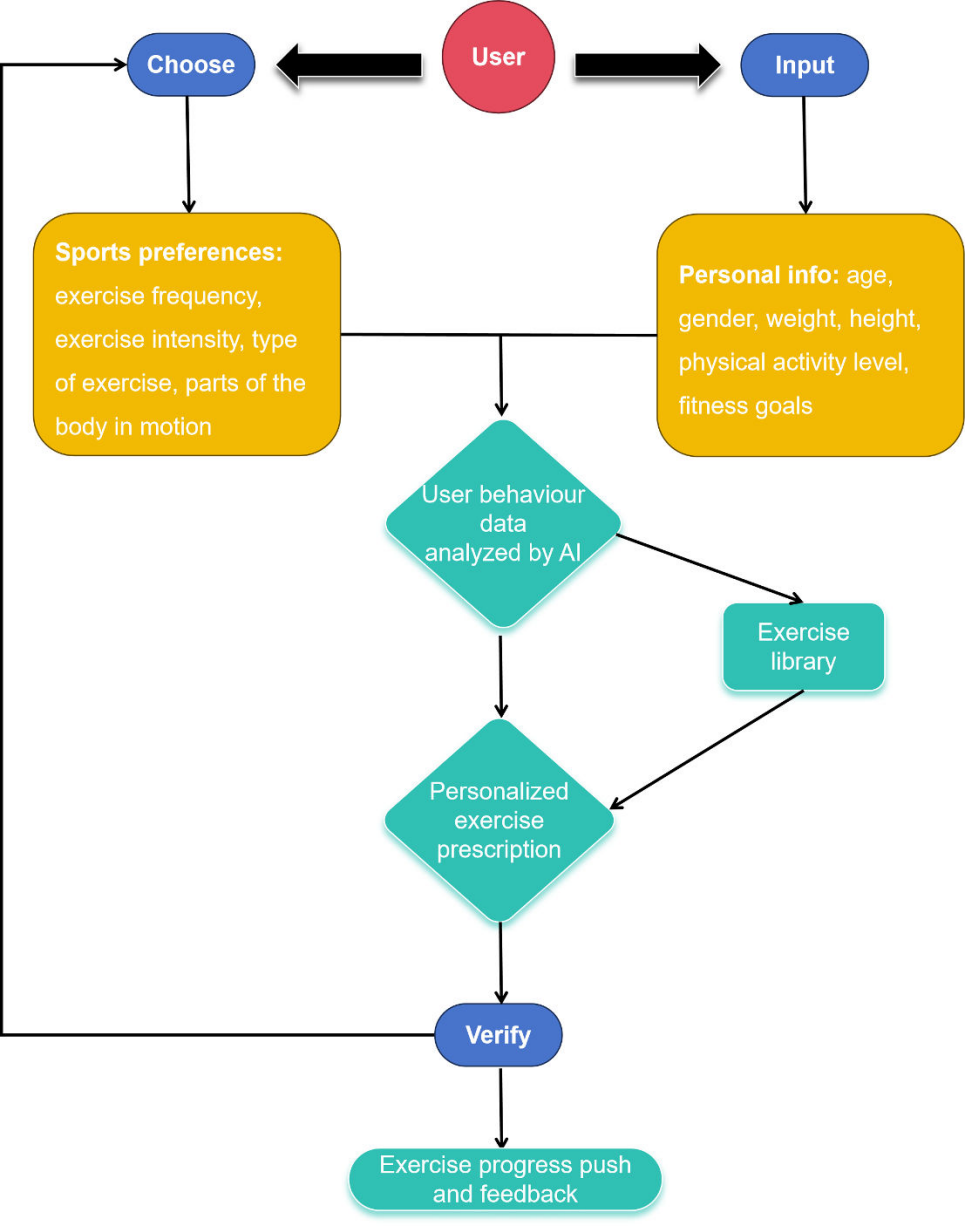
Personalized exercise prescription development. AI: artificial intelligence.

The app’s AI recommendation system analyses user data, including activity levels, preferences, and behavior patterns, to create highly personalized exercise prescriptions (Figure 3). The exercise library in the ShouTi Fitness app features a diverse selection of activities targeting various body parts, ranging from moderate to vigorous PA (3‐10 metabolic equivalent of energy [METs), with an average MET value of 7 METs (Multimedia Appendix 1).

##### Action Recognition

**Figure 4.**
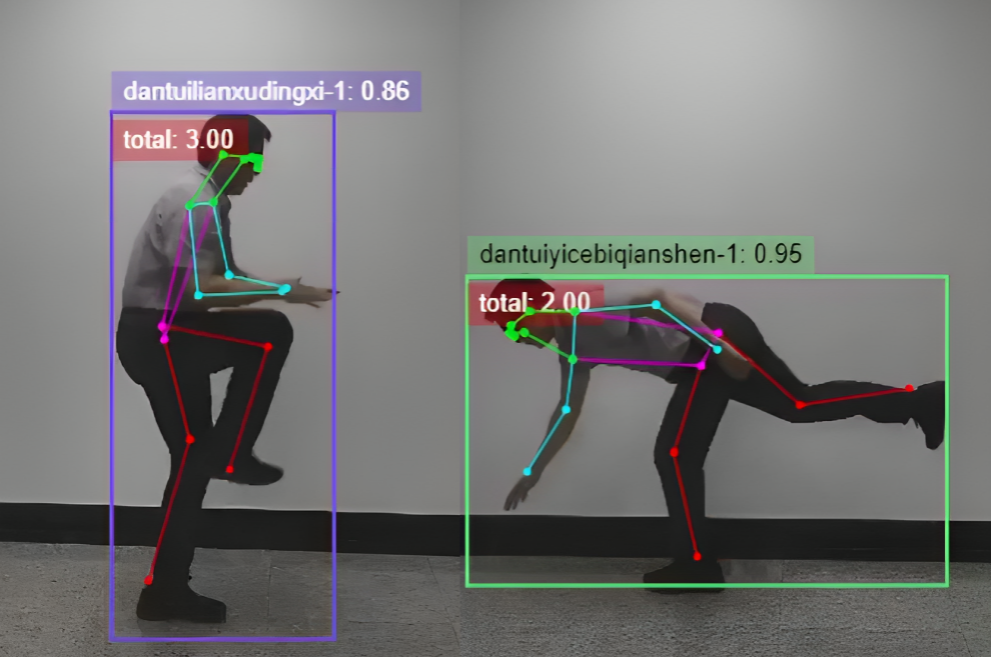
Artificial Intelligence motion recognition and monitoring.

The ShouTi Fitness app uses AI technology to provide real-time feedback and movement recognition (Figure 4). Its aim is to enhance the interactivity and accuracy of users’ exercise routines. The app functions by using motion recognition algorithms to monitor the user’s movements and posture throughout workouts, offering immediate feedback. This prompt correction assists users in adjusting their form in real time, thereby improving the effectiveness of their workouts and reducing the risk of performing exercises incorrectly, which could otherwise lead to injury.

##### Smart Grouping

AI plays a central role in creating balanced, optimized teams for the gamification. Using a combination of random assignment and a matching algorithm, participants are grouped into teams of 5 (Figure 2C). The AI analyzes a range of user characteristics, including age, gender, height, weight, PA level, and fitness goals, to create teams that are comparable in terms of physical capacity and fitness objectives. The algorithm uses a K-means clustering method to optimize team composition by minimizing intra-team differences based on users’ characteristics, ensuring that the competition remains fair and engaging for all participants:


$$D_{ij}=\sqrt{\sum_{k=1}^{n}{\left(x_{ik}-x_{jk}\right)}^{2}}$$

where $D_{ij}$ is the distance between participants i and j based on their characteristics.

##### Dynamic Management

The main function of dynamic management is to reset weekly rankings, match participants with new competitors, and dynamically adjust the competitive environment to keep the challenge fresh and motivating. At the end of each week, the AI calculates a team’s total score by aggregating the individual performances of all 5 members:


$$\;Total\;Score\;=\sum_{i=1}^{5}\left(\;PA\;Frequency{\;}_{i}+\;PA\;Duration{\;}_{i}\right)$$

By resetting weekly scores and rematching competitors, it ensures a fair and balanced competitive environment that encourages consistent effort, sustained engagement, and long-term success.

##### Team Collaboration and Competition

**Figure 5.**
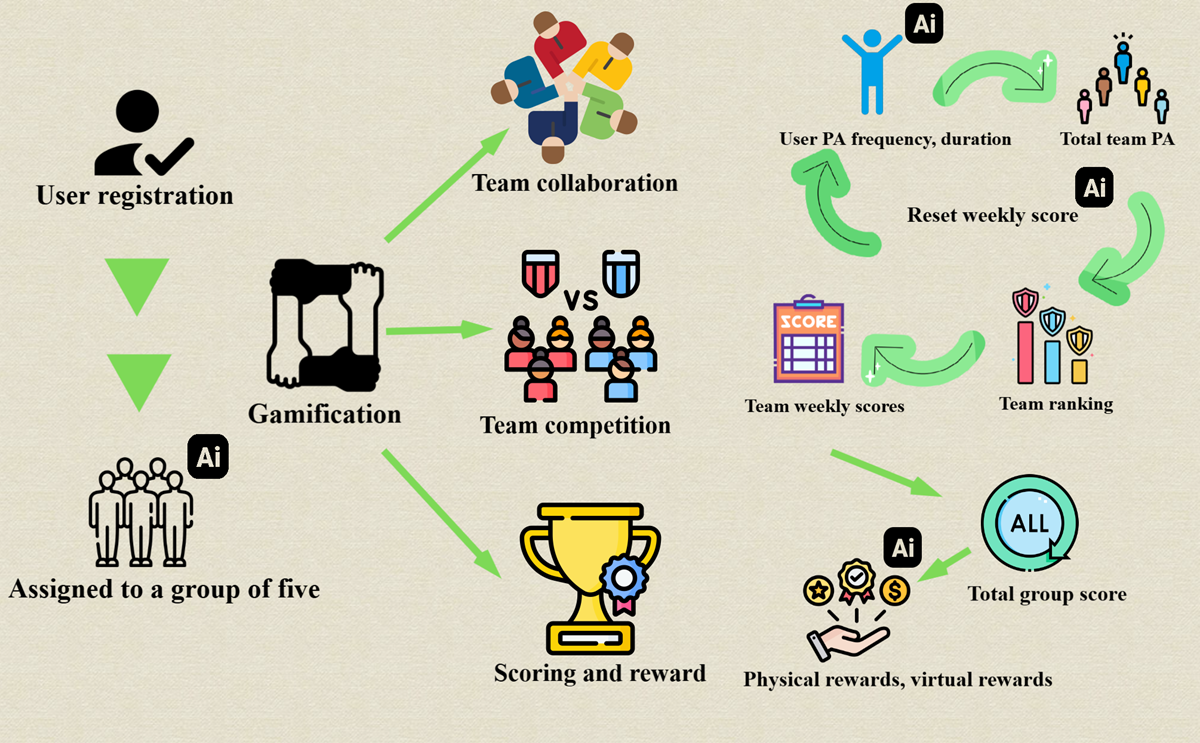
Artificial intelligence—powered gamification development. Note: The artificial intelligence functions involved in the diagram are: artificial intelligence-based smart grouping; artificial intelligence-based personalized exercise recommendation; artificial intelligence-based visual action recognition and feedback; artificial intelligence-based gamification intervention dynamic management. AI: artificial intelligence; PA: physical activity.

Collaboration and competition are central to the gamification experience (Figure 5). Teams are not only created with balanced composition, but also engaged in friendly competition through weekly rankings. At the end of each week, the AI assigns points to the top-performing teams based on their accumulated scores, with the highest-ranking teams earning rewards (Figure 2D). The competition fosters a sense of community, as users work together within teams to achieve collective fitness goals. The reward system includes both virtual achievements, such as badges and unlockable content, and tangible physical prizes, further incentivizing engagement and consistent effort across the app’s user base.

### Measures

#### Demographic Characteristics

At baseline, participants completed an online form within the app to provide their demographic information. This included age, gender, school, residence, height, weight, PA levels, and fitness goals.

#### Objective Physical Activity Measurement

PA was recorded using the ShouTi Fitness app, which logged the start time, end time, and duration of each PA session with high precision, down to the nearest second. This detailed data collection ensured accurate tracking of participants’ activity levels and allowed for precise measurement of their engagement with the exercise program. Descriptive statistics were used to summarize PA session counts and durations. Given the nonnormal distribution of the data, assessed using the D’Agostino test for normality, the median IQR were reported. The IQR is provided from the 25th to the 75th percentile. Percentages were used for categorical data. Scatter plots visualized the usage characteristics of ShouTi Fitness throughout the intervention period.

#### Usability and Acceptability of the AI-Powered Gamification Intervention

To evaluate the usability and acceptability of the AI-powered gamification intervention, participants completed an online survey within the app at follow-up. The survey included the System Usability Scale (SUS) and additional self-reported questions. The SUS, consisting of 10 items with responses ranging from strongly agree to strongly disagree, was scored on a 0‐100 scale [27]. Scores were calculated by summing the converted values and multiplying by 2.5. Based on Bangor et al’s [28] cutoffs, SUS scores were categorized as follows: excellent (85.58‐100), good (72.75‐85.57), OK (52.01‐72.74), and poor (0‐52.00). Additional questions assessed participants’ views on the intervention’s usefulness, their willingness to continue using it, and their likelihood of recommending it. Participants also provided feedback on technical issues and their engagement with the intervention’s features, including the AI technology and gamification elements.

#### Feasibility and Adherence of the AI-Powered Gamification Intervention

The study primarily focused on assessing the feasibility and adherence to the AI-powered gamification intervention by analyzing the correlation between PA session duration and the overall intervention duration. While this measure provides insights into user engagement over time, further assessments are necessary to directly evaluate the intervention’s effectiveness in increasing PA levels. The adherence effectiveness of the intervention was evaluated through Spearman rank correlation analysis, examining the relationship between the duration of individual PA sessions and the length of the intervention.

#### Characteristics of Participation: Gender Differences

To investigate gender differences in intervention participation characteristics, Mann-Whitney U tests were conducted on PA patterns for different genders. These analyses aimed to identify usage trends and preferences, providing insights for optimizing the intervention’s features and user-engagement strategies.

### Data Analysis

Statistical analyses were conducted using SPSS (version 26.0, IBMCorp) for data processing and GraphPad Prism for graphical representations. A significance level of *P*<.05 was used for all tests to determine statistical significance.

### Ethical Considerations

Eligible individuals received informed consent forms that outlined the study’s purpose, potential risks, and benefits. Consent was obtained both in person and through email, and participants provided their contact information for follow-up purposes. The study adhered to ethical guidelines, reviewed by the Academic Committee of Capital Institute of Physical Education (approval number: 2024A063), with informed consent obtained from all participants, who were fully briefed about the study and their rights. Participant confidentiality was strictly maintained throughout the research.

## Results

### Participant Activity Overview

This study involved 456 college students aged 18 to 25 years old, including 239 men (52.4%) and 217 women (47.6%). Over the 2-month study period, a total of 18,073 online PA sessions were recorded, all involving moderate or vigorous PA (3‐10 METs), with men contributing 8068 sessions and women 10,055 sessions. On average, men participated in 15 PA sessions (median) over the 2 months, while women participated in 22 sessions. The average duration of a single PA session was 66 seconds for men and 42 seconds for women (Table 1).

**Table 1.** Participant characteristics.

	Total (n=456), median (IQR)	Men (n=239), median (IQR)	Women (n=217), median (IQR)
Sessions	18 (6-39.5)	15 (5-39)	22 (9-40.5)
Duration (seconds)	52 (22-111)	66 (33-139)	42 (13-93)

### Usability and Acceptability of the Gamified AI App

Table 2 presents the usability and satisfaction results for the AI-powered gamification intervention. The average SUS score was 65.2, reflecting an “ok” usability rating. Of the 456 participants, 58 (13%) rated the experience as excellent, 197 (43%) rated it as good, 183 (40%) rated it as okay, and 18 (4%) rated it as poor, with the majority giving positive feedback. Regarding the intervention’s effectiveness in helping users achieve their fitness goals, 205 out of 456 participants (45%) strongly agreed and 159 out of 456 participants (35%) agreed, while only 24 out of 456 participants (5%) disagreed, showing a general consensus on its effectiveness. Most participants were satisfied with the AI functions: 191 out of 456 participants (42%) were very satisfied, 173 out of 456 participants (38%) were satisfied, 68 out of 456 participants (15%) were neutral, and 24 out of 456 participants (5%) were dissatisfied, reflecting strong approval of the intervention’s tailored plans.

**Table 2.** Participant ratings and satisfaction with the artificial intelligence—powered gamification intervention.

System Usability Scale score rating for intervention	Values, n (%)
**System Usability Scale score for the intervention**	
Excellent	58 (13%)
Good	197 (43%)
Ok	183 (40%)
Poor	18 (4%)
**The intervention helped me achieve my fitness goals**	
Strongly agree	205 (45%)
Agree	159 (35%)
Neutral	68 (15%)
Disagree	24 (5%)
**Be satisfied with the artificial intelligence functions**	
Strongly agree	191 (42%)
Agree	173 (38%)
Neutral	68 (15%)
Disagree	24 (5%)
**Would continue to use the intervention in future**	
Strongly agree	228 (50%)
Agree	137 (30%)
Neutral	68 (15%)
Disagree	23 (5%)
**Would recommend the intervention to others**	
Strongly agree	201 (44%)
Agree	164 (36%)
Neutral	68 (15%)
Disagree	23 (5%)
**Rate the reliability of this intervention**	
Excellent	160 (35%)
Good	205 (45%)
Ok	68 (15%)
Poor	23 (5%)
**Be attracted by the artificial intelligence features (eg, personalized recommendations, action recognition, smart grouping, and dynamic management)**	
Strongly agree	182 (40%)
Agree	159 (35%)
Neutral	68 (15%)
Disagree	45 (10%)
**Be motivated by the gamification elements (eg, team collaboration, team competitions, and rewards)**	
Strongly agree	219 (48%)
Agree	146 (32%)
Neutral	68 (15%)
Disagree	23 (5%)

Regarding future use, 228 out of 456 participants (50%) strongly agreed, and 137 out of 456 participants (30%) agreed that they would continue to use ShouTi Fitness for similar interventions, indicating good potential for continued use.The likelihood of recommending the intervention was also high, with 201 out of 456 participants (44%) strongly agreeing and 164 out of 456 participants (36%) agreeing, indicating users find it valuable. In terms of reliability, 160 out of 456 participants (35%) rated the intervention as excellent, 205 out of 456 participants (45%) rated it as good, 68 out of 456 participants (15%) rated it as okay.

The AI features, such as personalized recommendations, action recognition, smart grouping, and dynamic management, were appealing to 182 out of 456 participants (40%) who strongly agreed and 159 out of 456 participants (35%) who agreed. Gamification elements, such as team collaboration, team competitions and rewards, were well-received, with 219 out of 456 participants (48%) strongly agreeing and 146 out of 456 participants (32%) agreeing that these features were motivating, highlighting their effectiveness in boosting user engagement and motivation.

### Feasibility and Adherence of the AI-Powered Gamification Intervention

The distribution of daily PA sessions among the 456 students throughout the intervention period is shown in Multimedia Appendix 2, with a breakdown by gender shown in Multimedia Appendix 3. The frequency of daily PA sessions varied over time, with an initial increase followed by a decrease.

A Mann-Kendall test was used to analyze the time series data and the resulting trend curve is shown in Multimedia Appendix 4. The analysis showed that participants increased their daily PA sessions during the first 30 days of the intervention. However, from day 35 a significant decrease in PA frequency was observed, which continued until the end of the intervention. This pattern was consistent for both male and female participants.

Table 3 presents the results of the Spearman rank correlation analysis, which examined the relationship between the duration of each PA session and the total duration of the intervention. The analysis revealed a significant positive correlation (ρ=0.464, *P*<.001), suggesting that participants tended to increase the duration of their individual PA sessions as the intervention progressed. This suggests that the intervention was effective in increasing PA session adherence over time.

Notably, the correlation was stronger for male participants (ρ=0.510, *P*<.001) compared with female participants (ρ=0.258, *P*=.046). This pattern supports the idea that the intervention was more effective in promoting sustained PA engagement among men.

**Table 3.** Time-series correlation of the duration of each physical activity session.

Physical activity sessions	Spearman coefficient	*P* value
Total (N=18,073)	0.464	<.001
Men (n=8068)	0.51	<.001
Women (n=10,055)	0.258	.046

### Characteristics of Participation: Gender Differences

MultimediaAppendices 5  6 provide an insight into the usage patterns of the AI-powered gamification intervention among college students. The data show that men engaged in PA mainly in the dawn (23.4%), followed by the afternoon (21.8%), and the forenoon (20.3%). In contrast, women were most active in the afternoon (29.4%), followed by the forenoon (21.5%), and dawn (21.1%). Overall, the afternoon proved to be the peak time for PA, with minimal participation at dusk and in the evening (0.1% and 0.3% respectively). In addition, both men (94.%) and women (97.2%) showed a strong preference for short PA sessions of 1‐10 minutes, with longer sessions less common. This suggests a clear preference for short PA sessions among students. A significant gender difference was observed in the duration of a single PA session (Z=29.585, *P*<.001). Men exhibited longer PA durations than women, indicating a significant difference in PA patterns between genders when using the intervention.

## Discussion

### Principal Findings

This study assessed the feasibility and usability of an AI-powered gamification intervention, designed to improve PA levels among Chinese college students. Results showed that the intervention was effective in engaging users, particularly through its gamification elements and AI features. While overall usability and satisfaction were high, the intervention’s impact on PA frequency diminished after the first 30 days, suggesting the need for strategies to sustain long-term engagement. Notably, gender differences in activity patterns were also observed, underscoring the necessity for tailored interventions to address diverse user preferences.

Over the 2-month intervention period, the average PA intensity was 7 METs per session. Participants engaged in the intervention an average of 18 times, with men participating 15 times and women 22 times. Most participants preferred afternoon sessions and favored short-duration sessions, with men averaging 66 seconds per session and women 42 seconds.

### AI-Powered Gamification Intervention

Gamification has long been recognized as an effective strategy for increasing user engagement and enhancing app effectiveness [29]. Previous research consistently shows that smartphone-based gamification can significantly boost PA participation rates by providing motivation through rewards, competition, and goal setting [20,30-33]. The integration of AI with gamification, as exemplified by the ShouTi Fitness app, offers innovative opportunities to further enhance user motivation and engagement. In this study, the incorporation of AI (eg, personalized exercise recommendations, action recognition, smart grouping, and dynamic management) and gamification elements (eg, team competition, rewards, and leaderboards) played a pivotal role in motivating users.

The gamification elements, particularly team competition, rewards, and leaderboards, significantly boosted participants’ accountability and fostered a sense of achievement [29]. Weekly team matches created both a competitive and collaborative atmosphere, where users could cooperate within their teams while striving to outperform other teams. This dynamic structure, supported by real-time team rankings, allowed users to track their performance and compare it with others, reinforcing the idea that individual contributions impacted the team’s overall success. Rewards earned by reaching specific milestones, provided an extra layer of motivation, further promoting sustained engagement. During the first month, these gamification elements could contribute to longer PA session durations and increased session frequency, supporting the Achievement Goal Theory as a useful framework for understanding how gamification features drive motivation [34].

The combination of AI-powered personalization and gamification principles significantly contributed to the success of this intervention. Specifically, AI provides tailored feedback and challenges based on user data. This level of customization made the PA experience more relevant and engaging [35-37]. Users were more likely to maintain regular PA as the intervention continuously aligned with their fitness levels and preferences. Furthermore, AI-based visual simulation technology, which offers interactive content, holds great potential for further boosting engagement [38]. By providing an immersive and dynamic experience, this technology can sustain user interest and motivate continued participation. However, despite the positive reception of AI features, around 10% of participants reported that some recommendations felt repetitive or not sufficiently tailored to their fitness levels. This feedback highlights a critical area for improvement, refining the personalization algorithms to ensure that all recommendations remain fresh, relevant, and appropriately challenging.

### Challenges in Long-Term Engagement

Despite the initial success, the observed decline in PA frequency after 35 days points to the challenges of maintaining long-term engagement in gamified AI interventions. This decline in activity may be due to several factors. First, the novelty of the intervention may have initially led to high engagement, but as this novelty wore off, users’ interest likely declined [39]. In addition, the appeal of the intervention’s challenges may fade as users become familiar with its features, while the absence of continuous incentives may reduce motivation. The competitive elements of gamification, while initially effective, can also lead to fatigue, especially when combined with privacy concerns or social overload [40]. The SUS score of 65.2 indicates that the app’s overall usability fell short of expectations for a highly user-friendly application. Several factors may have contributed to this result. User feedback highlighted issues such as app stability and occasional malfunctions in AI features, which likely impacted the overall usability score. Technical disruptions can disrupt the seamless user experience and contribute to user dissatisfaction [41]. In addition, the app’s interface and usability may not have fully aligned with the preferences of all users, especially those less familiar with technology-driven fitness interventions.

To combat this decline, regular updates to challenges and goals based on user feedback could reignite interest. Exploring innovative gamification techniques such as interactive storylines or augmented reality could help maintain engagement. In addition, regular reminders, special events, and seasonal challenges could help maintain user interest and enthusiasm over time. For the long-term success of the intervention, regular updates of the app are necessary to introduce new features and ensure compatibility with evolving platforms.

### Strengths and Limitations

This study has several strengths.: It is the first to explore the integration of AI and gamification in a mobile health app to enhance PA, offering novel insights into how these technologies can boost PA among college students. With 456 participants from 18 universities in Beijing, the large sample size ensures diverse representation and improves the generalizability of the findings. The use of objective data to measure PA sessions ensures a reliable, bias-free assessment of user engagement, enhancing the study’s validity. The detailed analysis of intervention patterns, including time of day, session duration, and gender differences, provides insights into user preferences that can inform future interventions.

However, this study also has limitations. The 2-month intervention period may not be sufficient to assess the long-term sustainability of PA behaviors. The decline in PA frequency after 35 days suggests that a longer intervention period might yield more insights into long-term effectiveness and user retention. While the AI-powered gamification effectively promoted initial engagement, the decline after day 35 highlights the need for strategies to maintain long-term adherence. Although the study indicates a positive trend in PA adherence, it does not assess the health effectiveness or long-term impact on physical health or fitness outcomes. Further research is needed to evaluate the broader health-related effectiveness of the intervention.

### Conclusions

The AI-powered gamification intervention shows promise for improving PA levels among college students. While it demonstrates strong initial engagement and positive feedback, addressing challenges related to long-term engagement, app stability, and refinement of AI algorithms is essential to maximize its potential. Continued research into optimizing these features, along with a focus on long-term PA adherence, will be critical to improving the effectiveness of the intervention and ensuring sustained user engagement in future interventions.

## Supplementary material

10.2196/65498 Multimedia Appendix 1 Exercise movement library.

10.2196/65498 Multimedia Appendix 2 Distribution of daily physical activity frequency during the intervention.

10.2196/65498 Multimedia Appendix 3 Distribution of daily physical activity frequency by gender.

10.2196/65498 Multimedia Appendix 4 Trend plot of daily physical activity sessions and intervention.

10.2196/65498 Multimedia Appendix 5 Daily physical activity frequency patterns.

10.2196/65498 Multimedia Appendix 6 Duration of single physical activity session.

## Abbreviations

AI: artificial intelligence
MET: Metabolic equivalent of energy
PA: physical activity
SUS: System Usability Scale
