# Supplementary material for: Feasibility and Usability of an Artificial Intelligence—Powered Gamification Intervention for Enhancing Physical Activity Among College Students: Quasi-Experimental Study
Source: JMIR Serious Games. 2025 Mar 24;13:e65498. doi: 10.2196/65498 (PMC11957469; doi:10.2196/65498)
Supplement: Multimedia Appendix 1 [file games-v13-e65498-s001.pdf]

Multimedia Appendix 1: Exercise Movement Library

1. Exercise movement library interface in the application:

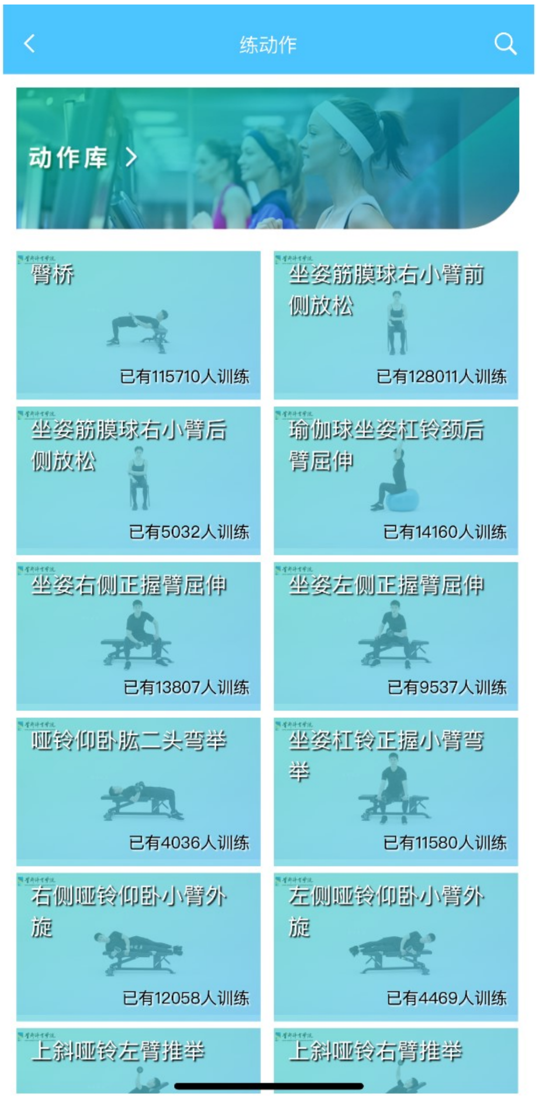

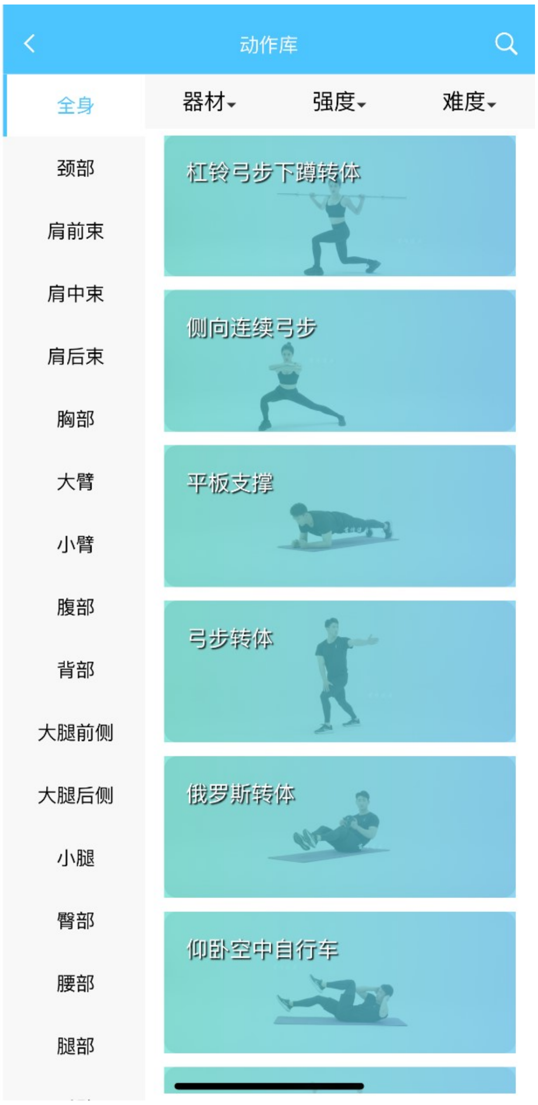

Over 100 exercises are included in the exercise movement library and they involve all parts of the body.

2. Exercise movement library example actions：

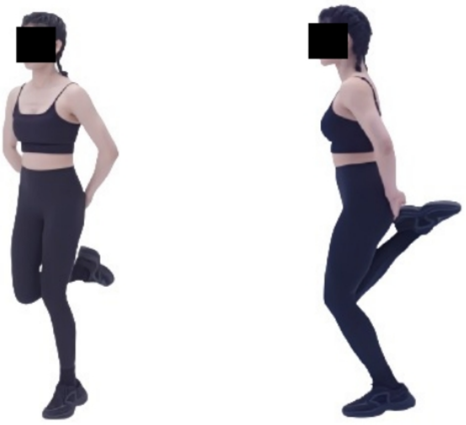

Butt kicks

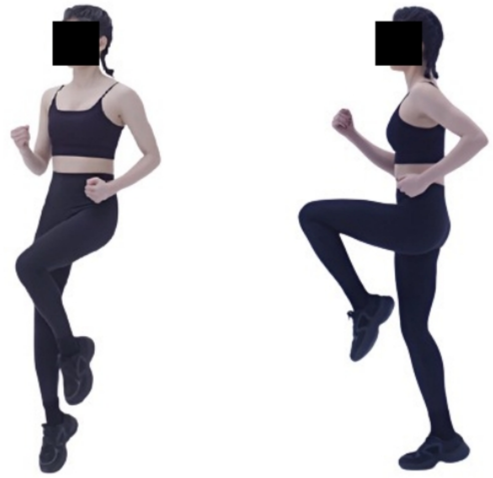

High knees

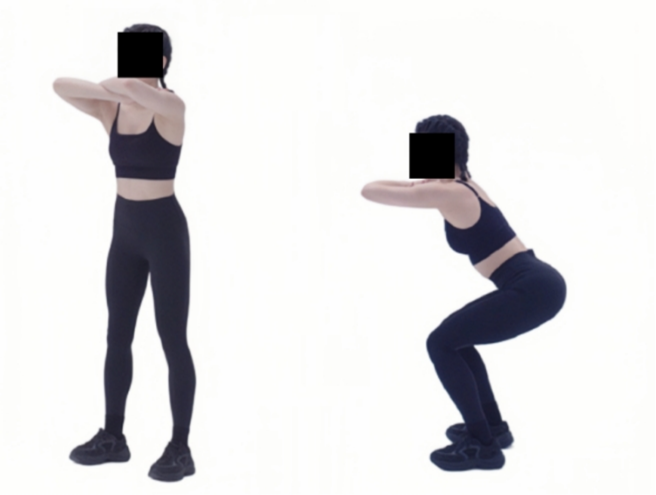

Squat up

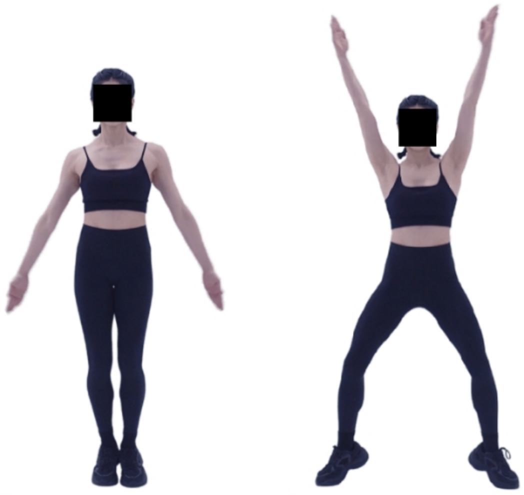

Jumping jacks

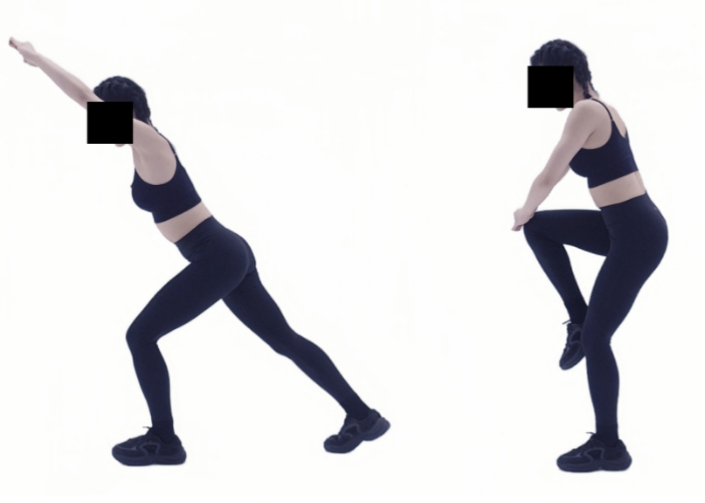

Single leg knee tucks

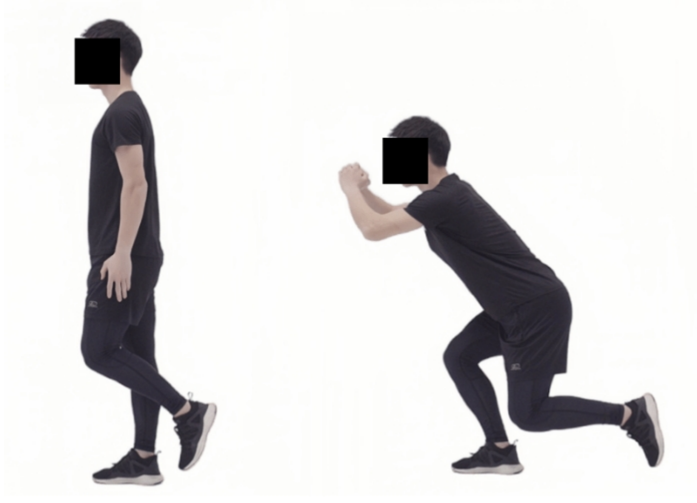

Single-leg squat

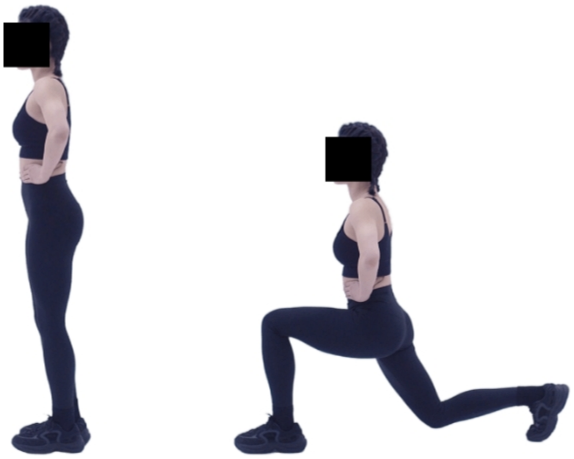

Forward lunge

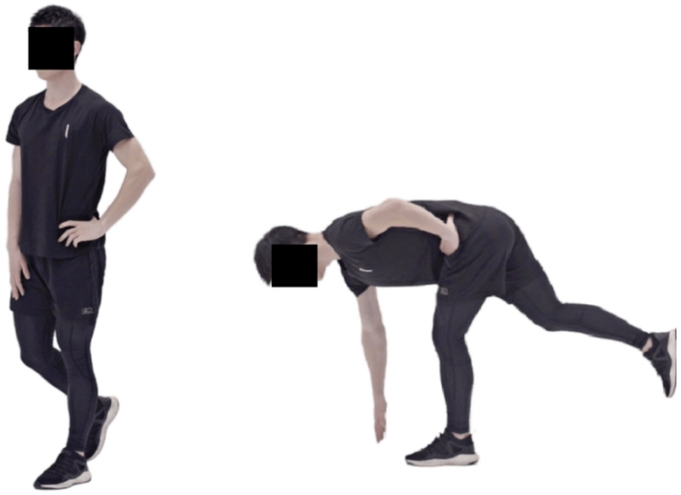

Single leg stiff-leg deadlift

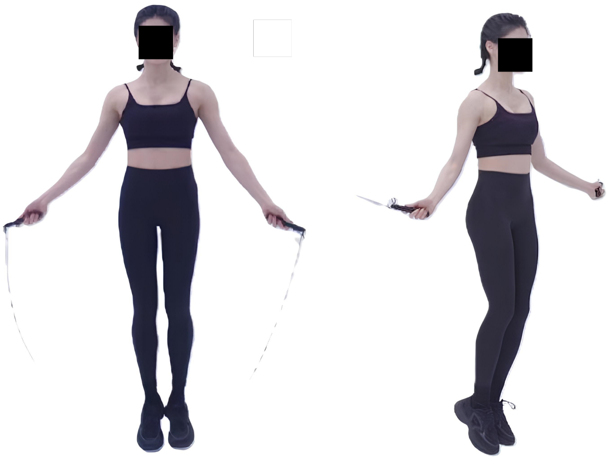

Jump rope

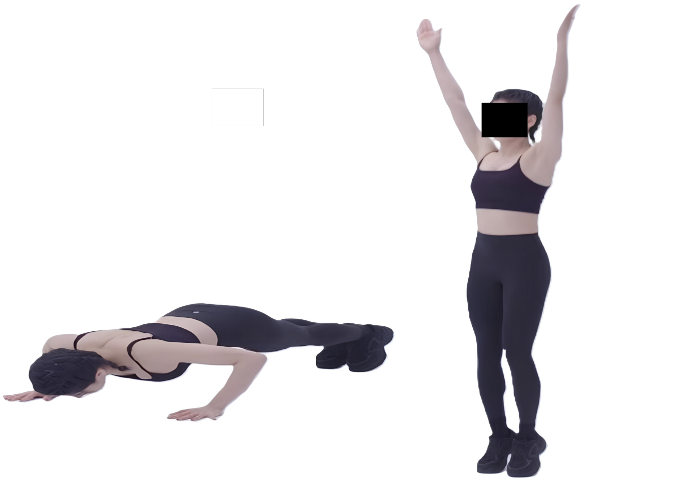

Burpee
