# Supplementary figures and images for: Feasibility and Usability of an Artificial Intelligence—Powered Gamification Intervention for Enhancing Physical Activity Among College Students: Quasi-Experimental Study
Source: JMIR Serious Games. 2025 Mar 24;13:e65498. doi: 10.2196/65498 (PMC11957469; doi:10.2196/65498)

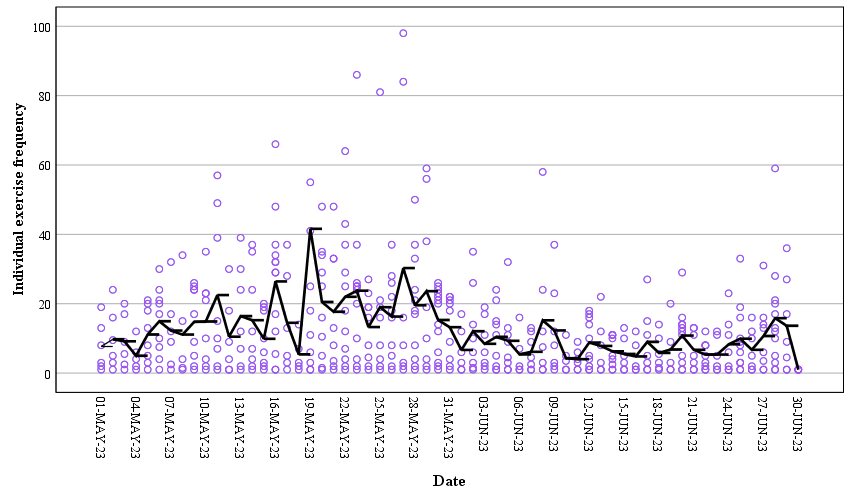

Supplement: Multimedia Appendix 2 [file games-v13-e65498-s002.png]

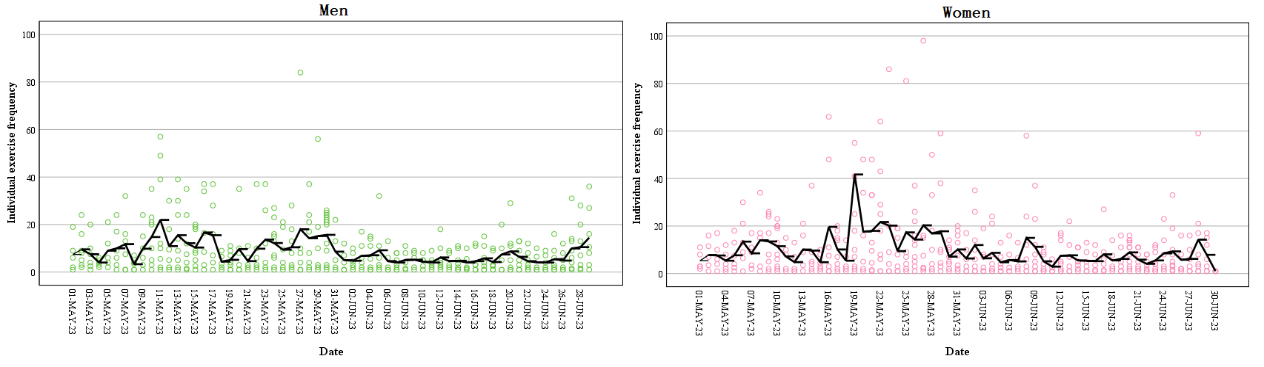

Supplement: Multimedia Appendix 3 [file games-v13-e65498-s003.png]

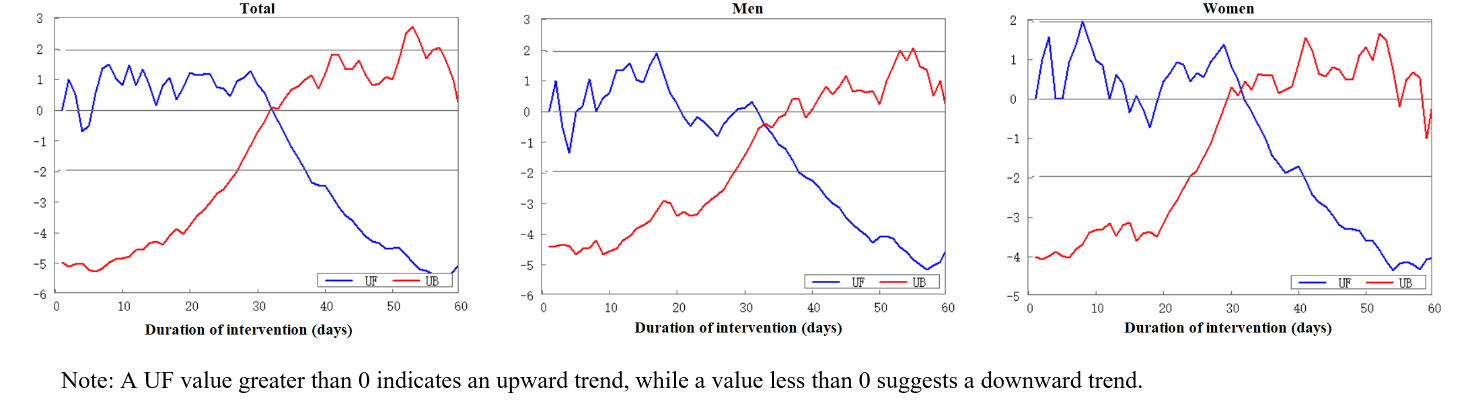

Supplement: Multimedia Appendix 4 [file games-v13-e65498-s004.png]

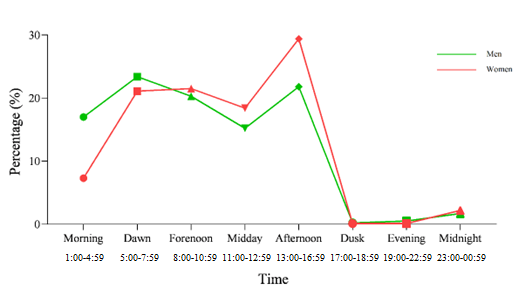

Supplement: Multimedia Appendix 5 [file games-v13-e65498-s005.png]

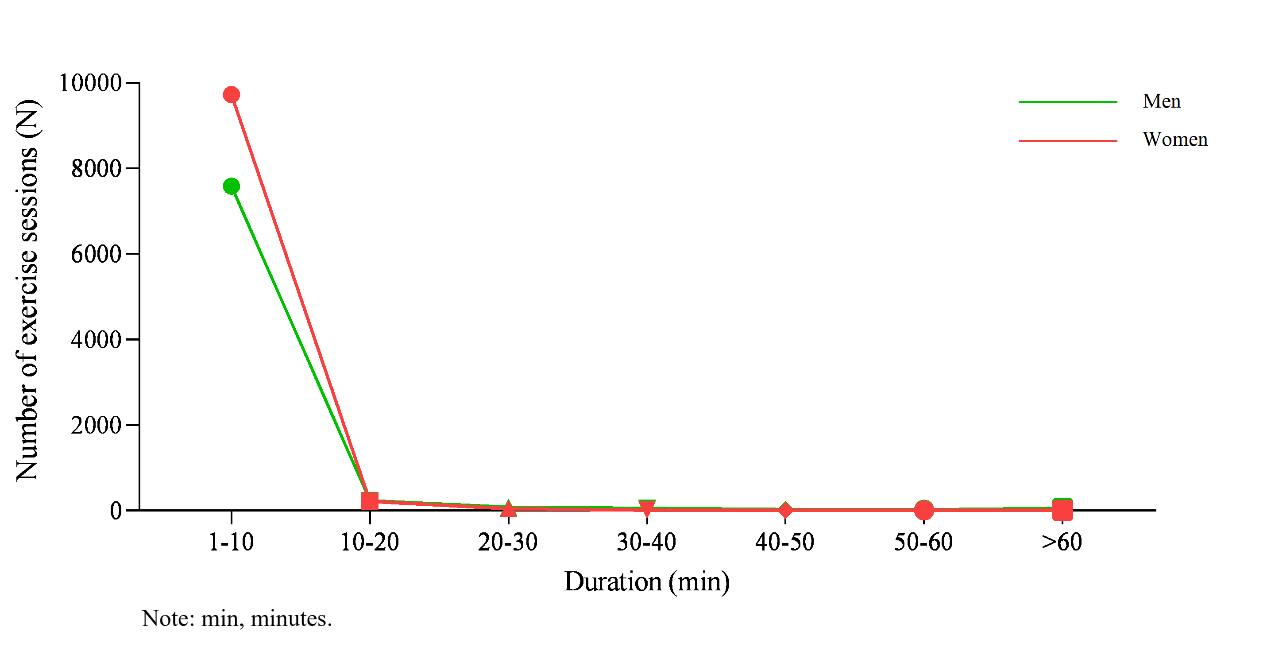

Supplement: Multimedia Appendix 6 [file games-v13-e65498-s006.png]
